# Hypertensive attack induced by dexamethasone during induction of anesthesia in a patient with an adrenal pheochromocytoma: a case report

**DOI:** 10.1186/s40981-022-00547-y

**Published:** 2022-08-06

**Authors:** Shuichiro Kurita, Yoshinori Kamiya

**Affiliations:** Department of Anesthesiology, Niigata University Medical and Dental Hospital, 1-754, Asahimachidori, Chuo-Ward, Niigata City, Niigata Prefecture 951-8510 Japan

**Keywords:** Pheochromocytoma, Hypertensive attack, Dexamethasone

## Abstract

**Background:**

Dexamethasone is used perioperatively as an antiemetic for postoperative nausea and vomiting. Evidence and mechanism linking dexamethasone and hypertensive attack of pheochromocytoma during anesthesia have not been reported.

**Case description:**

We report a case of a hypertensive attack during anesthetic induction immediately after dexamethasone administration in a 35-year-old woman with adrenal pheochromocytoma. Approximately 2 min after the anesthetic drugs and dexamethasone were administered, her arterial blood pressure suddenly increased from 143/79 to 243/116 mmHg during manual mask ventilation. Since tracheal intubation had not been performed yet, dexamethasone could be a causative agent of hypertensive episodes. The surgery and anesthesia were uneventful. She was admitted to the intensive care unit to have her blood pressure controlled subsequently.

**Conclusions:**

Dexamethasone should be used with caution in patients with adrenal pheochromocytoma on account of the risk of hypertensive attacks.

## Background

Patients undergoing pheochromocytoma resection have substantial intraoperative hemodynamic instability despite treatment with a preoperative alpha-adrenergic blocker. Several drugs are known to precipitate adverse reactions in patients with pheochromocytoma, and glucocorticoids have been reported to cause hypertensive attacks after some time post their intravenous administration. However, there are no reports of dexamethasone provided as a perioperative antiemetic causing a hypertensive attack immediately after administration. We report a case of a hypertensive attack during anesthesia induction after dexamethasone injection in a patient with adrenal pheochromocytoma and discuss the possible mechanisms.

## Case presentation

A 35-year-old patient (weight, 56 kg; height, 161 cm) with a right adrenal pheochromocytoma was admitted to our hospital for right adrenalectomy. Two months before the operation, the patient had chest pain and CT showed a 6-cm right adrenal mass. Urine analysis revealed markedly elevated levels of noradrenaline (1334.1 μg/day; normal, 31–160 μg/day), and its metabolite normetanephrine (3.34 μg/day; normal, 0.1–0.28 μg/day), even though adrenaline (7.1 μg/day; normal, 3.0–41.0 μg/day) and its metabolite metanephrine (0.06 μg/day; normal, 0.04–1.0 μg/day) were within normal range. A 123I-metaiodobenzylguanidine scintigraphy was positive only in the right adrenal gland. Her grandfather, mother, and sister had thyroid cancer, and the patient was strongly suspected to have MEN type 2. The patient was diagnosed with pheochromocytoma, and laparoscopic right adrenalectomy was planned after the introduction of the oral alpha-blocker doxazosin for 6 weeks to manage her blood pressure and hypertensive attacks.

Arterial blood pressure before anesthetic induction was 143/79 mmHg in the operating room. After epidural anesthesia was performed at the level of Th8/9 without pain and complication, general anesthesia was induced by sequential administration of remifentanil 0.3 μg/kg/min, propofol 100 mg, rocuronium 50 mg, and fentanyl 300 μg, without any pain or obnoxious reflex observed in the patient. At the same time, a bolus dose of 6.6 mg dexamethasone was administered for prophylaxis of postoperative nausea intravenously. Her ABP suddenly increased to 243/116 mmHg during manual mask ventilation, approximately 2 min after she was administered these anesthetic drugs. Her HR was steady at 71 beats/min, and SpO_2_ was steady at 100% under 5 L/min of oxygen. We administered 2 mg nicardipine immediately to control the hypertension, and her ABP was suppressed to 100/52 mmHg 1 min after the injection of nicardipine. Tracheal intubation was performed easily using McGrath MAC video laryngoscope (Covidien, Tokyo) with a normal endotracheal tube (Internal diameter 7.0 mm) without the occurrence of hypertension. We inserted a central line through the right internal jugular vein after tracheal intubation. During surgery, phentolamine (3 μg/kg/min) was administered continuously to prevent hypertensive attacks. The blood pressure transiently increased to 160mmHg during a tumor section; however, except this time period, hypertension (systolic blood pressure > 160mmHg) did not occur during surgery. After surgery, she was admitted to the intensive care unit to have her blood pressure and blood glucose levels controlled accordingly. In the intensive care unit, her blood pressure and blood glucose levels were found to be stable.

## Discussion

We experienced a hypertensive attack just immediately after bolus administration of dexamethasone during induction of general anesthesia in a patient with diagnosed pheochromocytoma. Anesthetic management for patients with pheochromocytoma requires attention to hypertensive attacks, and several drugs that cause hypertensive attacks are already known. However, there have been no reports of hypertensive attacks with dexamethasone administered to prevent PONV. Dexamethasone used for preventing PONV is not an essential agent in anesthetic management, and we should avoid using dexamethasone in patients with pheochromocytoma if it has the potential to produce a hypertensive crisis.

Several medications commonly used in anesthesia should be avoided or used cautiously in patients with pheochromocytoma. Although dexamethasone is listed in a review article as an alert [1], limited reports are available on the use of dexamethasone under general anesthesia. In this case, we thought that this hypertensive attack was related to dexamethasone since the attack occurred immediately after dexamethasone administration in the patient who had received adequate antihypertensive control with doxazosin and for whom the invasive procedures for tracheal intubation had not been performed until then. Furthermore, we took the utmost care to prevent hypertensive attacks in this pheochromocytoma patient during induction of anesthesia and used sufficient anesthetic induction drugs and narcotic analgesics to prevent hypertension during tracheal intubation. When the hypertensive attack occurred, the end-tidal concentration of sevoflurane was 1.5% and the estimated blood concentrations of fentanyl and remifentanil were 6.8 ng/ml and 2.2 ng/ml, respectively, which was considered sufficient to suppress noxious stimuli by tracheal intubation and vascular pain due to rocuronium. With respect to the management of anesthesia, we should avoid drugs that induce sympathetic tone, catecholamine release, and histamine release; however, rocuronium does not cause autonomic effects or histamine release [2].

Due to the rarity of pheochromocytoma, most data on anesthetic management and perioperative outcomes have been reported in small case series. In the past 11 case reports [3], as mentioned, it took several hours from glucocorticoid administration to the onset of side effects, such as hypertension, abdominal pain, and acute coronary syndrome in patients. None of these events occurred during general anesthesia. In terms of glucocorticoid potency, 6.6 mg of dexamethasone was relatively high. In two cases in which glucocorticoids of equivalent or higher potency were used, it took several hours or more for the onset of attacks.

Whether the hypertensive attack is specific to our patient or not is controversial. MEN type 2 patients, due to higher phenylethanolamine-*N*-methyltransferase and tyrosine hydroxylase expression, have an adrenergic phenotype with higher rates of catecholamine biosynthesis [4]. Our patient is highly suspected to have a MEN2-related gene based on her symptoms and findings (thyroid medullary carcinoma and family history). Hence, MEN type 2 patients, like in our case, may be a group of patients who are prone to a hypertensive crisis even with appropriate antihypertensive treatment.

## Conclusion

The use of high doses of dexamethasone for the prevention of PONV might be effective [4]. However, dexamethasone should be used with caution in patients with adrenal pheochromocytoma on account of the risk of hypertensive attacks.

## Data Availability

Not applicable
